# A unique case of inverted Meckel’s diverticulum presented as an adult intussusception: a case report

**DOI:** 10.1093/jscr/rjad288

**Published:** 2023-05-20

**Authors:** Elias E Lahham, Bashaer I Iwaiwi, Silvia Halteh, Hala Khaled, Mohammad AlQadi

**Affiliations:** Faculty of Medicine, Al-Quds University, Jerusalem, Palestine; Faculty of Medicine, Al-Quds University, Jerusalem, Palestine; Faculty of Medicine, Al-Quds University, Jerusalem, Palestine; Faculty of Medicine, Al-Quds University, Jerusalem, Palestine; Department of General Surgery, Beit-Jala Hospital, Bethlehem, Palestine

**Keywords:** Meckel diverticulum, intussusception, small bowel obstruction

## Abstract

Although Meckel’s diverticulum (MD) is a relatively common asymptomatic gastrointestinal anomaly, an inverted MD is a rare entity that is challenging to diagnose prior to surgery and presents usually in the pediatric population with bleeding, anemia and abdominal pain. The most frequent adult presentation in non-inverted MD is intestinal obstruction, whereas bleeding and anemia are the most typical presenting complaints in inverted MD. Here, we report our experience with an adult female patient, who presented with 5 days duration of abdominal pain, nausea and vomiting. Imaging revealed signs of small bowel obstruction with bowel wall thickening in the terminal ileum and a double target appearance. This case describes a rare cause of adult intestinal intussusception because of inverted MD, which was successfully managed with surgery. The final pathology report confirms the diagnosis.

## INTRODUCTION

Meckel’s diverticulum (MD) is the most common congenital anomaly of the intestines. It was first described by the German anatomist Johann Meckel in 1809 as an omphalomesenteric duct remnant [1]. The ‘rule of twos’ is a useful mnemonic for the features of MD although the figures do have a broad range: it occurs in 2% of the population, measures about 2 inches (5 cm) in length, located ~2 feet (60 cm) from the ileocecal valve, may contain two types of tissue (gastric and pancreatic) and it is symptomatic in just 2% of the affected population mostly under the age of 2 years [2]. Hemorrhage and anemia are the most frequent presenting complaint in inverted MD. Others include intestinal obstruction, diverticulitis, Littre hernia or perforation [3, 4]. Adult intussusception because of an inverted MD is exceptionally rare [5]. Preoperative diagnosis of inverted MD is challenging because of overlapping clinical and imaging characteristics. Therefore, it is necessary for surgeons to early recognize its various clinical presentations. Whether diagnosed preoperatively or intraoperatively, surgery remains the mainstay of treatment. Our study presents an adult female presented with a sign of small bowel obstruction because of intussusception of inverted MD that was managed successfully with surgery. The aim of this report is to shed light on this rare entity and to alert surgeons to not oversight MD in the adult population.

## CASE PRESENTATION

**Figure 1:**
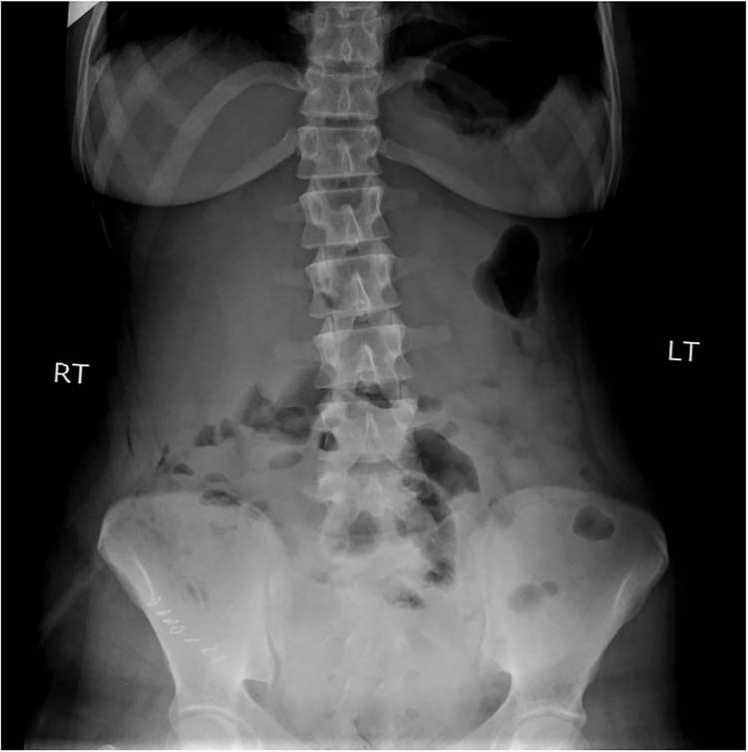
Plain abdominal X-ray demonstrated air-fluid levels of the small bowel.

**Figure 2:**
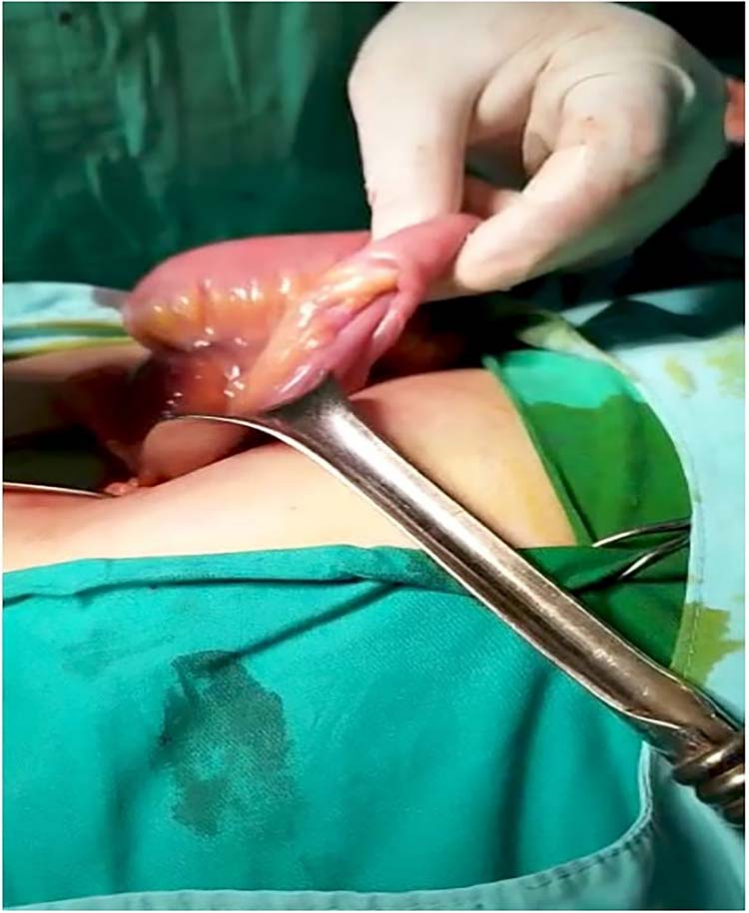
Intussuscepted portion of ileus attributed to inverted MD.

**Figure 3:**
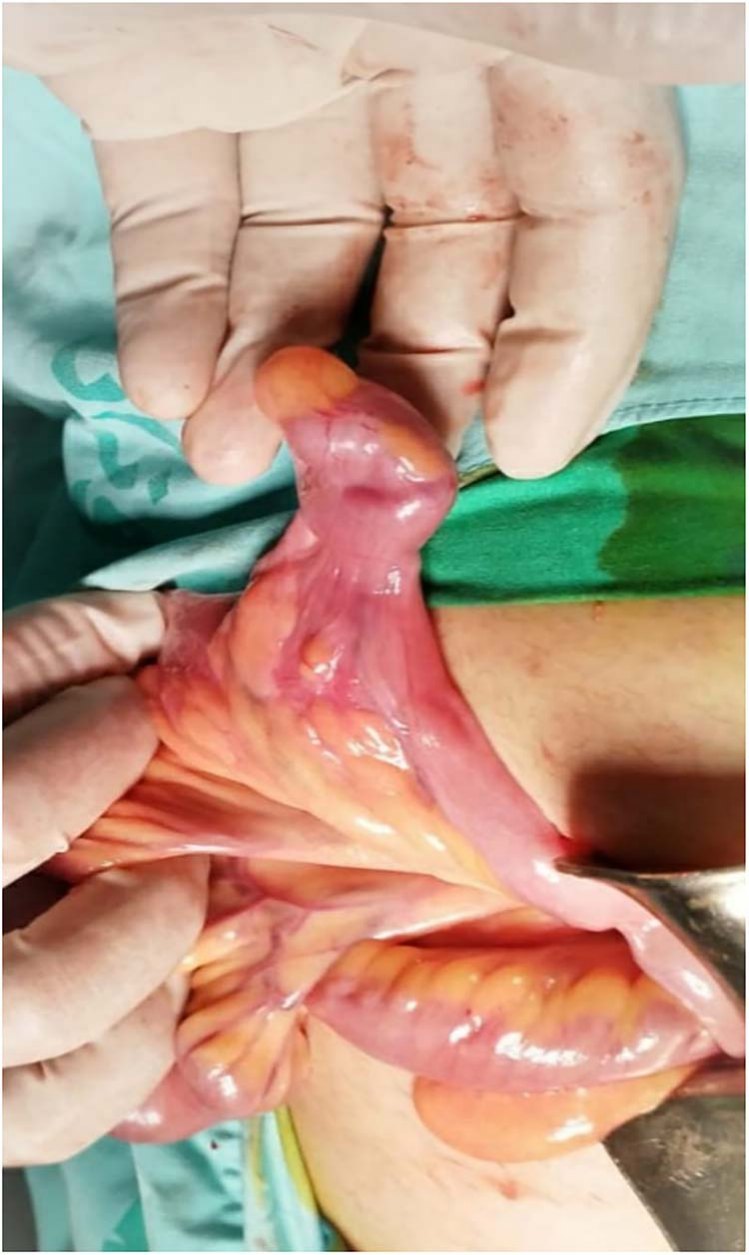
Intraoperative image of a free diverticulum located ~75 cm from the ileocecal valve.

**Figure 4:**
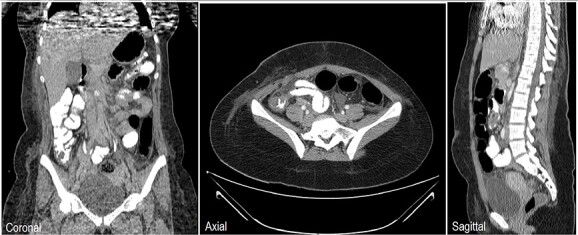
Chest, abdomen and pelvis CT scan with Oral & IV contrast postop Day 5 showed no contrast leak, no fistula and no obstruction at the site of surgery.

An 18-year-old female patient, with free past medical and surgical history, presented to the emergency department because of abdominal pain of 5-day duration associated with nausea, vomiting, constipation and loss of appetite. The pain was all over the abdomen mostly in the right lower quadrant, of gradual onset, colicky in nature, not radiated with any aggravating or relieving factors. It started intermittently and became continuous with increasing severity on the day of admission. On admission, she had normal vital signs. Physical examination showed a mildly distended abdomen with exaggerated bowel sounds. The patient had abdominal tenderness mostly in the right lower quadrant with abdominal guarding. Rovsing sign and rebound tenderness were both positive. However, the psoas and obturator signs were negative. No scars, hernias or masses were found. Laboratory tests are all within normal. A plain abdominal X-ray demonstrated air-fluid levels of the small bowel, with no pneumoperitoneum (Fig. 1**)**. Abdominal ultrasound showed bowel wall thickening in the ileum, a target-like mass containing a central area of increased echogenicity with a double target appearance. The initial diagnosis was a small bowel obstruction. Because of the severity of the abdominal pain, a decision to proceed with diagnostic laparotomy was made. Nasogastric decompression was done, IV fluids, antispasmodics and antibiotics were started. The patient underwent emergency surgery with a lower midline abdominal incision. Intraoperatively, an intussuscepted segment of the ileum measuring 8 cm in length located 75 cm from the ileocecal valve with dilated proximal ileal and jejunal loops was found and a soft polypoid shape mass was palpable within the lumen (Fig. 2*)*. Enterotomy was performed revealing a tubular segment measuring 6 × 2 × 0.8 cm with a globular swelling at the tip (Fig. 3*)*. No ischemic loop was identified. A diverticulectomy was performed in parallel with the longitudinal axon of the bowel lumen. Ectopic gastric mucosa was identified histologically confirming the diagnosis of inverted MD. The postoperative period was uneventful. CT scan with oral and IV contrast postoperative Day 5 showed no leak from the site of anastomosis *(*Fig. 4). The patient started a soft diet on Day 5 and was discharged on Day 7.

## DISCUSSION

MD occurs in 2% of the population [2]. The majority of cases appear in children under 2 years of age, and the incidence of MD complications decreases with advancing age. It is usually asymptomatic [2]. Gender distribution is four times more frequent in males. Furthermore, its complications are more common in males [6]. Up to 6.4% develop complications that need surgical intervention [7]. In adults, it accounts for <1% of causes of small bowel obstruction. Inverted MD is also rare, accounting for 4% of all cases presenting with intestinal obstruction because of intussusception [8]. The pathophysiology of inverted MD is not well established. However, the reasons for inversion include abnormal bowels peristalsis and the non-fixity of the diverticulum itself which acts as a leading point for the intussusceptions [3]. Several clinical findings including a history of previous episodes, painless rectal bleeding and possible palpable mass have been identified to distinguish the intussusception from an uncomplicated inverted MD. However, our patient did not have any of these typical characteristics. There is no gold standard diagnostic tool for an inverted MD causing an intussusception, some clinicians used abdominal ultrasonography as the first diagnostic modality for intussusception, which shows the classical target sign, whereas others encourage CT scan is the most useful tool to diagnose intussusceptions, provided that does not hamper surgical intervention [3]. Historically, Tc-99 m pertechnetate scintigraphy (Meckel’s scan) has been used as a diagnostic tool to detect ectopic gastric mucosa. However, in adults, it is a less reliable with a higher false negative rate and a specificity of just 9%, moreover, no ectopic tissue was found in 41% of patients with inverted MD, Therefore, a negative scan does not exclude the presence of MD [9]. Surgery is the mainstay of treatment for symptomatic MD. However, prophylactic surgical intervention in incidental MD still controversial [6]. Laparoscopy is safe and effective as an option in emergency surgery. However, we choose to do an open approach as the laparoscopic technique was not available in our center. Resection with anastomosis is clearly indicated in cases of inflammation and ischemia of the bowel, and edematous, inflamed or perforated base of MD [5]. However, diverticulectomy is a simple, minimal and cost-effective procedure that can treat the problem [10]. The decision to simple diverticulectomy vs. the resection with anastomosis of the small bowel should be individualized to each patient based on their clinical picture and pathology results.

## CONCLUSION

The diagnosis and treatment of inverted MD is a subject of debate because of its rarity and the limited number of reported cases in the literature. Regardless of the cause, when the diagnosis of adult intussusception is made, surgical intervention is mandatory. This case report aims to alert surgeons to this rare clinical entity and discuss the various circumstances under which inverted MD is managed. We recommend that any similar case to ours gets reported, to expand the subsequent knowledge of this entity.

## FUNDING

N/A.

## PATIENT CONSENT STATEMENT

Written informed consent was obtained from the patient for the publication of this case report and accompanying images. A copy of the written consent is available for review by the editor-in-chief of this journal on request.

## CONFLICT OF INTEREST STATEMENT

None declared.

## DATA AVAILABILITY

The data used to support the findings of this study are included within the article.
